# Ovulation-stimulation drugs and cancer risks: a long-term follow-up of a British cohort

**DOI:** 10.1038/sj.bjc.6605086

**Published:** 2009-05-12

**Authors:** I dos Santos Silva, P A Wark, V A McCormack, D Mayer, C Overton, V Little, J Nieto, P Hardiman, M Davies, A B MacLean

**Affiliations:** 1 Cancer Research UK Epidemiology and Genetics Group, Department of Epidemiology and Population Health, London School of Hygiene and Tropical Medicine, Keppel Street, London WC1E 7HT, UK; 2 Reproductive Medicine Unit, University College London Hospitals, Rowland Hill Street, London NW3 2PF, UK; 3 Department of Gynaecology, Royal Free Campus, Royal Free and University College Medical School, Huntley Street, London WC1E 6DH, UK

**Keywords:** infertility, clomiphene, gonadotrophins, ovarian stimulation, mortality

## Abstract

To assess long-term health effects of ovarian-stimulation drugs we followed-up for over 20 years a British cohort of 7355 women with ovulatory disorders, 43% of whom were prescribed ovarian-stimulation drugs, and identified a total of 274 deaths and 367 incident cancers. Relative to the general population, the cohort experienced lower mortality from most causes, including from all neoplasms combined, and lower incidence of cervical cancer, but higher incidence of cancers of the breast (relative risk: 1.13; 95% CI 0.97, 1.30) and corpus uteri (2.02; 1.37, 2.87). There were, however, no significant differences in the risk of cancers of the breast, corpus uteri, ovary, or of any other site, between women who had been prescribed ovarian-stimulation drugs and those who had not. Further analyses by type of drug and dose revealed a dose–response gradient in the risk of cancer of the corpus uteri (*P* for linear trend=0.03), with women given ⩾2250 mg of clomiphene having a 2.6-fold (2.62; 0.94, 6.82) increase in risk relative to those who were not treated. These findings do not support strong associations between ovulation-stimulation drugs and cancer risks, but they indicate the need for continued monitoring to establish whether risks are elevated in certain subgroups of users.

Several case reports in the 1980s, linking assisted conception and ovarian cancer, raised concerns about the long-term health effects of infertility treatment (Fishel and Jackson, 1989). These reports prompted many investigations into potential associations between exposure to fertility treatments used to stimulate ovulation and cancer risks. Some earlier studies (Whittemore *et al*, 1992; Rossing *et al*, 1994; Shushan *et al*, 1996) reported positive associations between ovarian stimulation and risk of ovarian cancer, but others did not confirm this. Raised risks have also been reported for cancers of the breast (Burkman *et al*, 2003; Orgéas *et al*, 2009), endometrium (Ron *et al*, 1987; Modan *et al*, 1998; Althuis *et al*, 2005; Calderon-Margalit *et al*, 2008), thyroid (La Vecchia *et al*, 1999) and malignant melanoma of the skin (Rossing *et al*, 1995; Calderon-Margalit *et al*, 2008), but again these findings were not replicated in other studies.

A cause–effect relationship between infertility treatment and cancer risks would have important implications for assisted conception programmes, which have been expanding considerably in recent years. A total of 34 855 women had *in vitro* fertilisation treatment in the United Kingdom in 2006 corresponding to 44 275 cycles of treatment, a 6.8% increase in the number of patients relative to the previous year (HFEA, 2008). We investigated the long-term health effects of the use of ovarian-stimulation treatments, with particular attention to potential cancer risks, in a large British cohort of women investigated for ovulatory disorders who have been followed-up for over 20 years.

## Materials and methods

Study subjects were identified through two case series of women who attended reproductive endocrinology practices in London. The first consisted of 7425 women who attended the Royal Free Hospital in 1963–1999 (Ginsburg and Hardiman, 1991). The second was assembled at University College Hospital (Reproductive Medicine Unit) and comprised 1727 women (including five also seen at the Royal Free Hospital), many of whom were treated with clomiphene citrate in the 1960s and 1970s as part of the initial evaluation of this drug, prior to it being available on the market. The present follow-up study was approved by all the relevant ethics committees.

From the meticulous clinical notes kept by the founders of these case series, a trained abstractor extracted and computerised relevant data, including information on signs and symptoms at presentation, final diagnosis, treatments prescribed (with number of cycles and dose) and their outcome. Hospital records (mainly on microfilms) and computer databases were also reviewed. Data on underlying diagnoses and treatments were reviewed by a panel of infertility clinicians and re-classified using more up-to-date and standardised criteria. Underlying ovulatory disorders were classified according to the WHO classification (WHO, 1973). For the specific purpose of this study, treatments were classified according to their physiological effects on the ovary and endometrium into five broader, and not-mutually exclusive, categories: (i) ovarian stimulation; (ii) ovarian physiological stimulation; (iii) ovarian suppression; (iv) endometrium stimulation; and (v) endometrium suppression (as detailed in Table 1).

Study subjects were followed through the National Health Service Central Register (NHSCR) in England and Wales to ascertain their vital status, and to obtain information on site-specific cancer incidence, cause-specific mortality and migrations. A total of 7444 women out of the initial 9152 (81.3%) were traced and flagged through this register. For 95% of the 1708 who could not be traced, there was insufficient detailed information (e.g., name too common with no information on exact date of birth) or the names were foreign suggesting that they might have been non-UK residents. A further 89 subjects were excluded because flagging was considered to be unreliable (*n*=8), they were no longer NHS patients at the time they joined the cohort (*n*=79), or they have subsequently undergone a sex change operation (*n*=2). For a further 183 women information on treatment was lacking and these were excluded from any treatment-related analyses. Women who were known to be still alive and resident in England and Wales, and whose current general practitioner (GP) could be traced through their Health Authorities, were sent (through their GPs) a postal questionnaire to obtain further details on their reproductive and lifestyle characteristics.

To compare mortality and cancer incidence with that in the general population, expected numbers of deaths and cancers in the cohort were calculated by applying England and Wales female rates to the number of person-years at risk, stratifying by 5-year calendar period and 5-year age bands. Time at risk for mortality analyses was estimated from the date of first hospital visit (or the date of first treatment for analysis by type of treatment) to death, emigration, loss to follow-up, or 31 December 2005, whichever occurred first. For cancer incidence analyses, time at risk was from 1 January 1971, when cancer registration reached national coverage, or from date of hospital visit/treatment if these occurred after that date, to the earliest of date of diagnosis of the first primary malignancy, emigration, loss to follow-up, date of death, or 31 December 2005. Period-age-standardised mortality (SMR) and incidence ratios (SIRs) were then calculated as the ratio between the observed and expected number of events (deaths or cancers, respectively) in the cohort, with their 95% percent confidence intervals (CI) estimated using an exact method (Sasieni, 1995).

Data on mortality and cancer incidence for England and Wales were provided by the Office for National Statistics as tabulations of numbers of female cancers and deaths by single calendar year, single year of age, and four-digit codes of the International Classification of Diseases (ICD) (revision 7 (ICD-7) for 1961–1967; ICD-8 for 1968–1978; ICD-9 for 1979–2000; and ICD-10 for 2001–2005) (WHO, 1957, 1967, 1977, 1994). Appropriate bridging of ICD codes across the various revisions was performed to ensure comparability throughout the follow-up period.

To account for socioeconomic differences between the cohort and the general population, we also obtained rates for England and Wales by quintiles of socioeconomic deprivation as defined by the Carstairs Index (Carstairs and Morris, 1989) and, from 1995 onwards, the income domain of the Index of Multiple Deprivation (IMD) (DETR, 2000). Rates for the top two quintiles (the two most affluent) of the national distribution of deprivation scores were used to estimate expected number of events in the cohort as, at that time, infertility treatment was sought mainly by women of high socioeconomic status (68.6% of the women who completed the questionnaire were in social classes I and II (the two most affluent)) (OPCS, 1991), based on their own or their partner's occupation, with only 5.1% being in social classes IV and V (the two least affluent); equivalent figures for England and Wales females were 27.8 and 23.1%, respectively (OPCS/GRO, 1993).

Two approaches were used to compare study groups within the cohort. In the first, the risk of dying from a particular cause, or of developing a certain site-specific cancer, among patients ‘exposed’ to a given characteristic (e.g., treatment type) relative to the risk among those ‘unexposed’ was estimated as the ratio between the two corresponding SMRs, or SIRs, to take into account calendar period and age effects. The 95% CI for these relative risks (RRs) were calculated using an exact method (Breslow and Day, 1987). The second approach used Cox proportional hazards models (Clayton and Hills, 1993) to examine treatment effects in more detail while adjusting for potential confounders. RRs were estimated as hazard rate ratios in users relative to non-users of a given treatment while adjusting for current age (as the analysis time-scale); other variables were included in the regression models to evaluate their roles as potential confounders or effect modifiers. Because of the small number of cases assessment of confounding was performed for each potential confounding variable one at a time, by comparing the age-adjusted and the age-variable-adjusted estimates within the subset of women with data on that variable. The proportional hazard assumption, evaluated by visual examination and a formal test (Schoenfeld, 1982; Grambsch and Therneau, 1994), was met for all models shown. All statistical analyses were performed using Intercooled Stata 10.0 (Stata Corporation, College Station, TX, USA).

## Results

The baseline characteristics of the women in the final study population (*n*=7355) were similar to those in the original cohort (*n*=9152) (Table 1). The mean age at presentation among the participants was 28.1 years, with a mean follow-up of 21.4 years; 89% of the participants were followed-up for at least 10 years and 14% for at least 30 years. Half of the participants presented because of menstrual disturbances (Table 1). After investigation, 24% were diagnosed with a WHO type II ovulatory disorder, mainly with polycystic ovarian syndrome. A total of 3196 (44.5%) patients received ovarian-stimulation treatments, 1976 receiving clomiphene only, with a median number of cycles equal to two corresponding to a median dose of 1000 mg per woman; 18% were prescribed more than the maximum of six cycles currently recommended by the Committee on Safety of Medicines (1995). A total of 1198 women were prescribed gonadotrophins (alone or in combination with clomiphene), with a median number of cycles per woman equal to three (Table 1).

Questionnaire data were available for 2545 participants (out of the 4475 who were still alive and whose current GP could be traced and was willing to forward the questionnaire to them; a response rate of 56.9%). There was no evidence that respondents differed from the remaining study population in terms of clinic attended (79.8 *vs* 83.8%, respectively, attended the Royal Free Hospital), age at initial clinical evaluation (mean±s.d.: 28.0±7.1 *vs* 28.5±8.4 years, respectively), or type of treatment (e.g., 29.6 *vs* 26.5% were prescribed clomiphene only, respectively).

In all, 274 deaths occurred during follow-up to the end of 2005; 47% were from malignant neoplasms, including 39 from breast cancer, 10 from ovarian cancer and 7 from cancer of corpus uteri (hereafter referred to as cancer of the uterus) (Table 2). Relative to the general population, mortality in the cohort was lower for all-causes (SMR=0.89), reflecting lower risks for most specific causes except, as expected, for endocrine and metabolic diseases (SMR=1.99) (Table 2). Analyses by cancer site showed increased mortality in the whole cohort for cancers of the liver and biliary tract (SMR=2.68) and of the uterus (SMR=3.02), with only the latter being statistically significant (Table 2); in contrast, mortality from cancers of the breast and ovary did not differ from those in the general population. Further analyses stratified by type of treatment revealed that the excess risk of dying from liver and biliary cancer was confined to women who were prescribed ovarian stimulation; these women had a four-fold increase (RR=4.19, based on small numbers; Table 2) in risk relative to those not given this treatment. Similarly, women who were given ovarian-stimulation drugs had more than two times (RR=2.37) the risk of dying from breast cancer than those who were not, with this difference being statistically significant. In contrast, those prescribed ovarian-stimulation drugs had only half the risk of dying from cancers of the uterus (RR=0.53) and ovary (RR=0.57) of those not given such drugs (Table 2), but these estimates were rather imprecise.

A total of 367 incident malignant neoplasms occurred from 1971–2005, comprising 177 breast, 31 uterine and 21 ovarian cancers. Relative to the general population, cohort members had increased risks of developing cancers of the breast (SIR=1.13), uterus (SIR=2.02) and nervous system (SIR=1.91), albeit only significant for the latter two, but a significantly lower risk of developing cervical cancer (SIR=0.21) (Table 3). Analyses stratified by type of treatment showed that, relative to the general population, women who were prescribed ovarian stimulation had a borderline raised risk of developing a malignant neoplasm (SIR=1.10), with significant increased risks for cancers of the breast (SIR=1.26) and uterus (SIR=2.31). There were also non-significant increased risks for cancers of the liver and biliary tract (SIR=2.59) and for nervous (SIR=1.96) and lymphatic and haematopoietic systems (SIR=1.66), as well as a significantly lower risk for cervical cancer (SIR=0.09). Women who were not prescribed ovarian stimulation had no increase in the risk of breast cancer (SIR=0.99), but a non-significant increased risk of cancer of the uterus (SIR=1.66); they also had a significantly lower risk of cervical cancer (SIR=0.36). Thus, risks among women who were prescribed ovarian stimulation relative to those not prescribed such treatments were not significantly raised for any cancer site although there were borderline increases for all neoplasms (RR=1.17) and cancer of the breast (RR=1.27) (Table 3). There were no associations between any of the other type of treatment categories (Table 1) and risks of cancers of the breast, uterus or ovary (data not shown).

The lower mortality in the cohort relative to the general population was mainly accounted by socioeconomic differences between the two populations as most SMRs became closer to unity when England and Wales rates for the two most affluent quintiles of the national distribution of area-based deprivation scores were used to derive expected numbers (e.g., the SMRs for deaths from circulatory, respiratory and digestive systems increased from 0.67, 0.77 and 0.87 (Table 2) to 0.96, 1.13 and 1.34, respectively); similarly, the magnitude of most SIRs increased slightly. However, the magnitude of the RRs associated with ovarian stimulation were little affected, with those for cancers of the breast, uterus and ovary being 1.27 (95% CI 0.93, 1.74), 1.40 (0.64, 3.18) and 1.40 (0.53, 3.96), very similar to those presented in Table 3.

Within cohort analyses do not suggest that associations between ovarian stimulation and risks for cancers of the breast and uterus were confounded by other risk factors for these cancers (small numbers precluded similar analyses for ovarian cancer). Having ever been pregnant as recorded in the clinical notes was not a confounder of the association of ovarian stimulation with cancers of the breast (age-adjusted RR=1.30 (95% CI 0.96, 1.77); age and ever-pregnancy-adjusted=RR 1.28 (0.94, 1.74)) or uterus (age-adjusted RR=1.53 (0.72, 3.23); age- and ever-pregnancy-adjusted RR=1.73 (0.81, 3.67)). Similarly, adjustment for having ever been pregnant before treatment, or for having become pregnant as a result of it, did not affect the magnitude of these cancer associations. In the subset of women who completed the questionnaire (comprising 41 treated and 28 untreated breast cancer cases and a total of 30 583 person-years of follow-up) the age-adjusted RR associated with ovarian stimulation was 1.02 (95% CI 0.63, 1.65) and its magnitude changed little with further adjustment for having ever been pregnant (1.02 (0.63, 1.66)), age at first pregnancy (1.03 (0.61, 1.75)), ever-use of oral contraceptives (1.01 (0.62, 1.64)) or hormone replacement therapy (1.05 (0.65, 1.71)), or a positive family history of breast cancer (1.05 (0.65, 1.72)). The small number of cases among respondents precluded similar analyses for cancer of the uterus.

Ovarian-stimulation treatment was associated with underlying diagnosis with, for instance, higher proportions of treated *vs* untreated women among WHO type II ovulatory disorders (53 *vs* 47%), but lower proportions among thyroid disorders (39 *vs* 61%) and weight-related problems (48 *vs* 52%). There were, however, no associations between underlying diagnosis and cancer risks except that women with type II ovulatory disorders had an elevated risk of cancer of the uterus (2.82 (1.13, 6.70)) relative to women with other diagnoses. Further adjustment for underlying diagnosis did not affect the magnitude of the ovarian stimulation – cancer of the uterus association (e.g., age-adjusted and age and WHO type II-adjusted RR: 1.53 (0.72, 3.23) and 1.48 (0.60, 3.63), respectively). There was also no evidence that the ovarian-stimulation effects were modified by any of these risk factors although the power of the study to detect interactions was limited. In particular, the magnitude of the associations with cancer risks was not modified by age when the treatment was first prescribed. Only 121 women developed ovarian hyperstimulation syndrome and the small numbers of cases among them (only one cancer of the breast and one of the uterus) precluded examination of whether risks were particularly elevated in this subgroup.

More detailed analyses by type of ovarian stimulation drug prescribed showed that the risk of developing breast cancer was significantly elevated among women who were prescribed clomiphene only (SIR=1.41), but this risk was not higher than that among women who were not treated (ovarian stimulation or any other) (Table 4). There were no clear dose–response trends in the risk of breast cancer with time since first treatment, total cumulative dose, or number of cycles of clomiphene, although women in the highest exposure categories had the highest risks (Table 4). The risks of cancer of the uterus were significantly raised among women who took ovarian-stimulation drugs but again none of the risks were significantly higher than those observed among the women not given any type of treatment. There were no clear trends in the risk of this cancer with time since first treatment, but there was a positive trend with total cumulative dose and number of cycles of clomiphene, with the first being significant (*P* for linear trend=0.034). Thus, women who took ⩾2250 mg were 2.62 (95% CI 0.94, 6.82) times more likely to develop cancer of the uterus than those who were not treated. The risk of cancer of the ovary was not associated with any of the ovarian-stimulation treatments, and there was no evidence of any trends in risk with time since first use, total cumulative dose or number of cycles of clomiphene, but estimates were based on small numbers. Risks for any of these three cancer sites were not associated with time since first treatment with gonadotrophins or number of cycles or ampoules, but again relevant numbers were small (not shown).

## Discussion

This cohort study has several strengths. Notably, in the absence of trials, cohort studies are the next best design because information on exposures is obtained prior to onset of disease. Linkage to the NHSCR ensured that information on cause-specific mortality and cancer incidence was also unbiased relative to the exposure status of the cohort members and minimised losses to follow-up. Second, the study had a long follow-up, allowing examination of long-term effects of ovarian stimulation. Third, it benefited from detailed information on causes of infertility and drug exposures from medical records as well as on cancer risk predictors obtained through completed questionnaires (although the latter only for a subset). Fourth, about one fifth of women were exposed to high levels of ovarian-stimulation drugs, well above currently recommended maximum levels. Fifthly, the availability of an internal comparison group may have minimised possible confounding by any factors correlated with treatment-seeking behaviour, because women who attended the two reproductive endocrinology practices are likely to differ from the general population in several respects. In particular, our study confirmed that women who seek infertility treatments tend to be healthier and of a higher socioeconomic background as those in the general population. Finally, the availability of information on underlying diagnosis and other risk factors also enabled adjustment for a variety of potential confounding factors.

Weaknesses of our study include the fact that follow-up was possible only for 80% of the original cohort; however, there was no evidence that those untraced through the NHSCR differed from those who were traced. Although the number of cancer cases accrued during the follow-up was larger than in most similar cohorts (Venn *et al*, 1995, 1999; Doyle *et al*, 2002; Klip *et al*, 2002), although not all (Brinton *et al*, 2004; Jensen *et al*, 2007; Calderon-Margalit *et al*, 2008: Jensen *et al*, 2009), the numbers for certain sites, particularly uterus and ovary, were too small to provide reliable estimates. The numbers will increase with increasing follow-up as these women are now reaching the ages when cancer risk sites are high. Follow-up for cancer incidence was possible only from 1971, but although we may have missed some earlier cases, most women were recruited later. Data on various potential confounding variables were available but its quality and completeness were not always ideal, being limited to women who were still alive and traceable at the time of the re-contact, hence residual confounding by these and other correlates of infertility cannot be excluded.

Our study found increased incidence of, and mortality from, breast cancer among women who were prescribed ovarian-stimulation treatments relative to those who were not, albeit the relative risk was significant only for mortality data. Further analyses by cumulative dose, and number of cycles did not reveal any clear dose–response gradients. The lack of such trends would argue against a true cause–effect relationship. This interpretation would be consistent with findings from most cohort (Modan *et al*, 1998; Venn *et al*, 1999; Klip *et al*, 2002; Doyle *et al*, 2002; Brinton *et al*, 2004) and case–control studies (Weiss *et al*, 1998; Ricci *et al*, 1999) that have assessed the relationship between fertility treatments and breast cancer risk, which have not found any overall associations, although some (Jensen *et al*, 2007; Orgéas *et al*, 2009) reported increases in specific subgroups.

This study provides no evidence that ovarian stimulation is associated with an elevated risk of ovarian cancer. In contrast, some earlier case series and epidemiological studies (Whittemore *et al*, 1992; Rossing *et al*, 1994; Shushan *et al*, 1996) reported positive associations, with risk being particularly high in the subgroup of nulligravid women, whereas among those who took drugs but did achieve a pregnancy the risk was not significantly different from that among gravid women without a history of infertility. More recent cohort (Modan *et al*, 1998; Venn *et al*, 1999; Klip *et al*, 2002; Doyle *et al*, 2002; Brinton *et al*, 2004; Calderon-Margalit *et al*, 2008; Jensen *et al*, 2009) and case–control (Ness *et al*, 2003) studies, however, have failed to find any such association. The lack of clear trends with time since start of treatment would also argue against the hypothesis that ovulation stimulation induces growth of pre-existing latent tumours.

There was some evidence that cancer of the uterus may be associated with ovarian stimulation, with risks increasing with increasing cumulative dose of clomiphene and, possibly, number of cycles. Few studies have examined this question but three large cohort studies reported increases among women exposed to high doses or with longer follow-up (Modan *et al*, 1998; Althuis *et al*, 2005; Calderon-Margalit *et al*, 2008). Such a link would be biologically credible as clomiphene and tamoxifen are both selective estrogen-receptor modulators and tamoxifen use has been shown to be associated with an increased risk of cancer of the uterus (Swerdlow *et al*, 2005). Although high doses of clomiphene may have been preferentially given to women with polycystic ovarian syndrome, a known risk factor for cancer of the uterus, adjustment for underlying diagnosis only slightly reduced the magnitude of the risk estimate.

Ovarian stimulation was not associated with colorectal cancer, malignant melanoma of the skin, or thyroid cancer. There was some evidence suggestive of a positive association with cancer of the liver and biliary tract, but this may be a chance finding (based on only three cases). Such a link has not been reported earlier but it has some biological plausibility as oral contraceptive use is known to be associated with increased risks of benign hepatic adenoma (Edmondson *et al*, 1976) and liver cancer (IARC, 1999).

The significantly lower risk of cervical cancer in this cohort relative to the general population is consistent with a possible surveillance bias and the fact that parity increases the risk of this cancer (ICESCC, 2006). There is some evidence in our study that risk may be somewhat lower among women who were exposed to ovarian stimulation than among those who were not (RR=0.24; Table 3), but the small number of cases precluded proper examination of dose–response effects.

Overall, the results of this study do not support strong associations between ovulation-stimulation treatments and cancer risks, with the exception of possible increases in the risk of cancers of the uterus and of the liver and biliary tract. These findings support the need for continuing monitoring of the long-term effects of these treatments.

## Figures and Tables

**Table 1:** Baseline and follow-up characteristics of the study population

	**Original cohort (*N*=9152)**		**Study population (*N*=7355)**	
**Characteristic**	***N*** a	**Statistics**	***N*** a	**Statistics**
*Follow-up*				
Calendar year of initial clinical evaluation (*n* (%))	9152		7355	
1949–1969		781 (8.5)		366 (5.0)
1970–1979		2853 (31.2)		2200 (29.9)
1980–1989		3957 (43.2)		3454 (47.0)
1990–1999		1561 (17.1)		1335 (18.2)
Age at initial clinical evaluation (mean±s.d.)	8915	27.9±7.9	7355	28.1±7.9
Years of follow-up to end of 2005 (mean±s.d.)	N/A	N/A	7355	21.4±8.3
Years of follow-up for cancer incidence analyses (1971–2005) (mean±s.d.)	N/A	N/A	7317	20.7±8.0
				
*Presentation symptoms*^b^ *(n (%))*	8666		7048	
Infertility for⩾1 year		3234 (37.3)		2532 (35.9)
Hirsutism		2097 (24.2)		1801 (25.6)
Menstrual disturbances		4379 (50.5)		3560 (50.5)
Weight related		582 (6.7)		462 (6.6)
Other^b^		853 (9.8)		727 (10.3)
				
*Underlying diagnosis (n (%))*	7743		6361	
WHO – group II^c^		1803 (23.3)		1517 (23.9)
Not WHO group II-related diagnoses		5940 (76.7)		4844 (76.2)
				
Most common specific diagnoses:^d^				
Polycystic ovarian syndrome (PCOS)		1775 (22.9)		1494 (23.5)
Male factor		566 (7.3)		489 (7.7)
Thyroid disease		541 (7.0)		423 (6.7)
Anovulation, irregular ovulation, primary amenorrhoea^e^		456 (5.9)		366 (5.8)
Weight-related ovulatory disorders		392 (5.1)		337 (5.3)
Hyperprolactinaemia		348 (4.5)		294 (4.6)
				
*Treatment prescribed (n (%))*	8876		7172	
Ovarian stimulation		3882 (43.7)		3196 (44.6)
Only clomiphene		2445 (63.0)		1976 (61.8)
Only gonadotrophins		243 ( 6.3)		190 (5.9)
Clomiphene+gonadotrophins		1168 (30.1)		1008 (31.5)
Physiological stimulation of ovary^f^		662 (7.5)		562 (7.8)
Suppression of ovary^g^		1150 (13.0)		984 (13.7)
Endometrium stimulation^h^		5178 (58.3)		4163 (58.1)
Endometrium suppression^i^		1364 (15.4)		1231 (17.2)
None of the above		2753 (31.0)		2176 (30.3)
*Ovarian stimulation: total dose & no. of cycles (median (25th, 75th percentile))* j				
Clomiphene – dose (mg/woman)	2662	1000 (350, 2100)	2241	1000 (350, 2250)
Clomiphene – no. of cycles/woman	3134	2 (1, 5)	2599	2 (1, 5)
Gonadotrophins – dose (number of ampoules/woman)	714	60 (27, 135)	643	60 (27, 138)
Gonadotrophins – no. of cycles/woman	717	3 (2, 6)	647	3 (2, 6)

a Number of women with information on this variable.

b Categories are not mutually exclusive. The category ‘other’ comprises galactorrhoea (study population=163), fertility untested (*n*=49), breast disease (*n*=29), history of recurrent abortion/miscarriages (*n*=1).

c In addition to PCOS patients, this category comprised women with Cushing's syndrome or congenital adrenal hyperplasia.

d Categories are not mutually exclusive. Other diagnoses in the study population: tubal disease (*n*=212), premature ovarian failure (*n*=134), history of past surgical intervention (*n*=103), endometriosis (*n*=75), cervical factor (*n*=70), pituitary tumour (*n*=48), congenital adrenal hyperplasia (*n* =30), hypogonadotrophic hypogonadism (*n*=29), gonadal dysgenesis (*n*=26), Cushing's syndrome (*n*=14), uterine factor (*n*=13), history of past benign ovarian tumour (*n*=12), secondary hypopituitarism (following neurosurgery or radiation: *n*=4), history of recurrent abortion/miscarriage (*n*=3), Turner's syndrome (*n*=2l).

e An additional 105 women in the original cohort and 89 in the study population were registered as being menopausal.

f Includes gonadotrophin-releasing hormones; bromocriptin and dopamine agonists.

g Includes inhibitors of gonadotrophins; combined oral contraceptives, male sex hormones and cyproterone acetate.

h Includes ovarian stimulation treatments, gonadotrophin-releasing hormones, bromocriptine, dopamine agonists, oestrogens and tamoxifen

i Includes inhibitors of gonadotrophins, progesterone, male sex hormones, androgen agonists (danazol, gestrinone) and cyproterone acetate

j Among users of any given drug only.

**Table 2:** Mortality in the cohort for selected disease categories, by type of treatment

		**All women (** ***n*=7355)**		**Women prescribed ovarian-stimulating drugs (** ***n*=3194)^a^**		**Women not prescribed ovarian-stimulating drugs (** ***n*=3976)^a^**		
**Cause of death**	**ICD-10**	**O/E^b^**	**SMR (95% CI)**	**O/E^b^**	**SMR (95% CI)**	**O/E^b^**	**SMR (95% CI)**	**RR (95% CI)^c^**
All causes	A00-Z99	274/306.5	0.89 (0.79–1.01)	132/141.9	0.93 (0.78–1.10)	134/157.7	0.85 (0.71–1.01)	1.09 (0.85–1.40)
All malignant neoplasms	C00-C85, C88-C94.3, C94.7-C97.9, D32, D33, D42, D43	126/143.3	0.88 (0.73–1.05)	69/70.0	0.99 (0.77–1.25)	54/70.1	0.77 (0.58–1.01)	1.28 (0.88–1.86)
Digestive system	C15-C26, C48	20/24.5	0.82 (0.50–1.26)	8/11.5	0.69 (0.30–1.37)	12/12.5	0.96 (0.50–1.68)	0.72 (0.26–1.92)
Colon and rectum	C18-C21	8/10.5	0.76 (0.33–1.50)	3/5.0	0.60 (0.12–1.76)	5/5.3	0.94 (0.30–2.19)	0.64 (0.10–3.31)
Liver and biliary tract	C22-C24	5/1.9	2.68 (0.87–6.25)	4/0.9	4.48 (1.22–11.47)	1/0.9	1.07 (0.03–5.95)	4.19 (0.41–206.48)
Respiratory system	C30-C39, C45	16/21.0	0.76 (0.44–1.24)	7/10.0	0.70 (0.28–1.44)	9/10.6	0.85 (0.39–1.61)	0.82 (0.26–2.48)
Malignant melanoma	C43	2/2.7	0.73 (0.09–2.63)	1/1.4	0.72 (0.02–4.03)	1/1.3	0.78 (0.02–4.32)	0.93 (0.01–73.11)
Breast	C50	39/40.3	0.97 (0.69–1.32)	28/20.4	1.37 (0.91–1.98)	11/19.0	0.58 (0.29–1.04)	2.37 (1.14–5.27)
Corpus uteri	C54, C55	7/2.3	3.02 (1.21–6.23)	2/1.1	1.81 (0.22–6.53)	4/1.2	3.42 (0.93–8.76)	0.53 (0.05–3.69)
Ovary	C56, C57.0-C57.4	10/11.6	0.86 (0.41–1.58)	3/5.8	0.52 (0.11–1.51)	5/5.6	0.90 (0.29–2.10)	0.57 (0.09–2.95)
Circulatory system	I00-I99	46/68.5	0.67 (0.49–0.90)	20/28.8	0.69 (0.42–1.07)	26/38.4	0.68 (0.44–0.99)	1.03 (0.54–1.91)
Respiratory system	J00-J99	17/22.0	0.77 (0.45–1.24)	11/9.3	1.19 (0.59–2.12)	5/12.3	0.41 (0.13–0.95)	2.92 (0.93–10.71)
Digestive system	K00-K93	15/17.2	0.87 (0.49–1.44)	4/8.2	0.49 (0.13–1.25)	10/8.6	1.16 (0.56–2.14)	0.42 (0.10–1.45)
Endocrine and metabolic	E00-E90	10/5.0	1.99 (0.95–3.66)	5/2.3	2.22 (0.72–5.18)	5/2.7	1.88 (0.61–4.39)	1.18 (0.27–5.13)
Nervous system, eye and ear	G00-G99							
	H00-H95	9/9.7	0.92 (0.42–1.75)	5/4.6	1.09 (0.35–2.54)	4/4.9	0.82 (0.22–2.09)	1.33 (0.29–6.70)

CI=confidence interval; SMR=standardised mortality ratio.

a Information on treatment missing for 183 women. Two additional women who were known to have been treated with ovarian-stimulating drugs were excluded from the analyses on treatment, because information on the start date of treatment was unavailable. Among these women eight deaths occurred during the follow-up.

b Number of observed (O) and expected (E) deaths estimated assuming the cohort experienced the same calendar year and age-specific mortality rates as the England and Wales female general population.

c Relative risk (95% confidence interval) associated with ovarian stimulation as estimated by the ratio between the two corresponding SMRs.

**Table 3:** Cancer incidence in the cohort for selected sites, by type of treatment

		**All women (***n*=**7317)^a^**		**Women prescribed ovarian-stimulating drugs (***n*=**3180)^a^**		**Women not prescribed ovarian-stimulating drugs (***n*=**3949)^a^**		
**Cancer**	**ICD-10**	**O/E^b^**	**SIR (95% CI)**	**O/E^b^**	**SIR (95% CI)**	**O/E^b^**	**SIR (95% CI)**	**RR (95% CI)^c^**
All	C00-C85, C88-C94.3, C94.7-C97.9, D32, D33, D42, D43	367/358.8	1.02 (0.92–1.13)	197/179.1	1.10 (0.95–1.26)	161/170.8	0.94 (0.80–1.10)	1.17 (0.94–1.45)
Digestive system	C15-C26, C48	29/41.2	0.70 (0.47–1.01)	15/19.8	0.76 (0.42–1.25)	14/20.6	0.68 (0.37–1.14)	1.11 (0.50–2.49)
Colorectum	C18-C21	17/25.1	0.68 (0.39–1.08)	11/12.2	0.90 (0.45–1.62)	6/12.4	0.48 (0.18–1.05)	1.87 (0.64–6.17)
Liver and biliary tract	C22-C24	3/2.4	1.25 (0.26–3.65)	3/1.2	2.59 (0.53–7.58)	0/1.2	0.00 (0.00–3.09)	5.05 (0.29–6254)
Respiratory system	C30-C39, C45	16/25.5	0.63 (0.36–1.02)	7/12.3	0.57 (0.23–1.17)	9/12.7	0.71 (0.32–1.35)	0.80 (0.25–2.42)
Bone and articular cartilage	C40, C41	4/2.8	1.42 (0.39–3.63)	1/1.4	0.74 (0.02–4.10)	3/1.4	2.17 (0.45–6.36)	0.34 (0.01–4.21)
Malignant melanoma of skin	C43	14/16.7	0.84 (0.46–1.41)	6/8.1	0.74 (0.27–1.60)	8/8.1	0.99 (0.43–1.95)	0.74 (0.21–2.44)
Breast	C50	177/157.3	1.13 (0.97–1.30)	102/80.7	1.26 (1.03–1.53)	72/72.6	0.99 (0.78–1.25)	1.27 (0.93–1.75)
Cervix uteri	C53	5/23.5	0.21 (0.07–0.50)	1/11.6	0.09 (0.00–0.48)	4/11.1	0.36 (0.10–0.92)	0.24 (0.00–2.43)
Corpus uteri	C54, C55	31/15.3	2.02 (1.37–2.87)	18/7.8	2.31 (1.37–3.64)	12/7.2	1.66 (0.86–2.90)	1.39 (0.63–3.16)
Ovary	C56, C57.0-C57.4	21/21.7	0.97 (0.60–1.48)	12/10.9	1.10 (0.57–1.93)	8/10.3	0.78 (0.34–1.53)	1.42 (0.53–3.99)
Urinary tract	C64-C68	5/9.3	0.54 (0.17–1.25)	1/4.5	0.22 (0.01–1.24)	3/4.6	0.65 (0.13–1.90)	0.34 (0.01–4.27)
Nervous system (incl. benign and unspecified), eye and ear	C69-C72, D32, D33, D42, D43	14/7.3	1.91 (1.05–3.21)	7/3.6	1.96 (0.79–4.03)	5/3.5	1.42 (0.46–3.30)	1.38 (0.38–5.52)
Thyroid and other glands	C73-C75	6/5.1	1.18 (0.43–2.57)	4/2.4	1.66 (0.45–4.25)	2/2.5	0.79 (0.10–2.86)	2.10 (0.30–23.2)
Lymphatic and haematopoietic system	C81-C85, C88, C90.0-C90.2, C91.0-C91.4, C91.5-C94.3, C94.6-C94.9, C96.0-C96.3, C96.7, C96.9	32/22.9	1.40 (0.95–1.97)	14/11.1	1.26 (0.69–2.12)	17/11.2	1.51 (0.88–2.42)	0.83 (0.38–1.80)

CI=confidence interval; SIR=standardised incidence ratio.

a Numbers differ slightly from those shown in Table 2 because of deaths, cancer incidence and migrations prior to 1 January 1971 when national follow-up for cancer incidence began. Information on treatment was missing for 182 women eligible for analyses on incidence, and information on the start date of treatment was missing for an additional six women: these women were excluded from the analyses on treatment.

b Numbers of observed (O) and expected (E) incident cancers estimated assuming the cohort experienced the same age and calendar year-specific cancer incidence rates as the England and Wales female general population.

c Relative risk (95% confidence interval) associated with ovarian stimulation as estimated by the ratio between the two corresponding SIRs.

**Table 4:** Risks of cancers of the breast, corpus uteri, and ovary by type of ovarian-stimulation treatment, time since first treatment, and dose

	**Cancer of breast (***n*=**174)**			**Cancer of corpus uteri (***n*=**30)**			**Cancer of ovary (***n*=**20)**		
	**O/E^a^**	**SIR (95% CI)**	**RR^b^ (95% CI)**	**O/E^a^**	**SIR (95% CI)**	**RR^b^ (95% CI)**	**O/E^a^**	**SIR (95% CI)**	**RR^b^ (95% CI)**
*Type of treatment* c									
No treatment	45/41.1	1.10 (0.80–1.47)	1 (baseline)	6/4.0	1.50 (0.55–3.27)	1 (baseline)	3/5.7	0.52 (0.11–1.53)	1 (baseline)
Ovarian stimulation	102/80.7	1.26 (1.03–1.53)	1.15 (0.80–1.68)	18/7.8	2.31 (1.37–3.64)	1.53 (0.58–4.72)	12/10.9	1.10 (0.57–1.93)	2.10 (0.57–11.60)
Clomiphene only	66/46.8	1.41 (1.09–1.79)	1.29 (0.87–1.92)	10/4.5	2.23 (1.07–4.11)	1.49 (0.49–4.98)	7/6.3	1.11 (0.44–2.28)	2.11 (0.48–12.63)
Gonadotrophins only	5/5.3	0.94 (0.31–2.20)	0.86 (0.27–2.16)	1/0.5	1.93 (0.05–10.75)	1.28 (0.03–10.59)	0/0.7	0.00 (0.00–5.19)	1.34 (0.00–26.52)
Both	31/28.4	1.09 (0.74–1.55)	1.00 (0.61–1.61)	7/2.8	2.51 (1.01–5.16)	1.67 (0.48–6.01)	5/3.8	1.31 (0.43–3.06)	2.50 (0.49–16.09)
									
*Time since first treatment (years)* *Super-ovulation*									
None	72/72.6	0.99 (0.78–1.25)	1 (baseline)	12/7.2	1.66 (0.86–2.90)	1 (baseline)	2/1.8	1.10 (0.13–3.96)	1 (baseline)
<10	9/12.9	0.70 (0.32–1.32)	0.70 (0.31–1.41)	2/0.7	2.80 (0.34–10.12)	1.69 (0.18–7.58)	5/4.0	1.24 (0.40–2.89)	1.41 (0.15–7.05)
10–19	44/31.6	1.39 (1.01–1.87)	1.40 (0.94–2.07)	7/2.5	2.85 (1.15–5.88)	1.72 (0.57–4.73)	5/5.0	1.00 (0.32–2.33)	1.59 (0.41–5.51)
⩾20	49/36.2	1.35 (1.00–1.79)	1.36 (0.93–1.99)	9/4.6	1.94 (0.89–3.68)	1.17 (0.43–3.02)	2/1.8	1.10 (0.13–3.96)	1.28 (0.33–4.44)
*P for linear trend*^d^			*0.15*			*0.46*			*0.84*
									
* Clomiphene only*									
None	77/78.2	0.98 (0.78–1.23)	1 (baseline)	13/7.8	1.67 (0.89–2.86)	1 (baseline)	8/11.0	0.73 (0.31–1.43)	1 (baseline)
<10	6/7.0	0.85 (0.31–1.86)	0.87 (0.31–1.98)	0/0.4	0.00 (0.00–9.33)	0.76 (0.00–8.32)	1/1.0	0.97 (0.02–5.40)	1.33 (0.03–9.96)
10–19	25/18.2	1.38 (0.89–2.03)	1.40 (0.85–2.22)	6/1.4	4.34 (1.59–9.44)	2.59 (0.81–7.31)	3/2.3	1.28 (0.26–3.74)	1.77 (0.30–7.36)
⩾20	35/21.6	1.62 (1.13–2.25)	1.65 (1.07–2.48)	4/2.7	1.48 (0.40–3.80)	0.89 (0.21–2.87)	3/3.0	1.01 (0.21–2.97)	1.40 (0.24–5.83)
*P for linear trend*^d^			*0.15*			*0.58*			*0.95*
									
*Total cumulative dose (mg)*									
*Clomiphene (all)*									
None	77/78.2	0.98 (0.78–1.23)	1 (baseline)	13/7.8	1.67 (0.89–2.86)	1 (baseline)	4/3.0	1.34 (0.36–3.42)	1 (baseline)
<900	28/22.4	1.25 (0.83–1.81)	1.27 (0.79–1.98)	2/2.0	0.98 (0.12–3.53)	0.58 (0.06–2.58)	4/2.4	1.65 (0.45–4.23)	1.84 (0.41–6.88)
900–2249	19/17.4	1.09 (0.66–1.71)	1.11 (0.63–1.85)	4/1.8	2.22 (0.61–5.70)	1.33 (0.32–4.30)	1/2.4	0.41 (0.01–2.29)	2.28 (0.50–8.50)
⩾2250	27/17.8	1.52 (1.00–2.20)	1.54 (0.95–2.41)	8/1.8	4.39 (1.89–8.64)	2.62 (0.94–6.82)	8/11.0	0.73 (0.31–1.43)	0.57 (0.01–4.23)
		*P for linear trend* d	*0.50*			*0.034*			*0.34*
									
*Number of cycles*									
*Clomiphene (all)*									
None	77/78.2	0.98 (0.78–1.23)	1 (baseline)	13/7.8	1.67 (0.89–2.86)	1 (baseline)	8/11.0	0.73 (0.31–1.43)	1 (baseline)
1–3	24/18.6	1.29 (0.83–1.92)	1.31 (0.79–2.10)	2/1.7	1.18 (0.14–4.28)	0.71 (0.08–3.13)	3/2.5	1.20 (0.25–3.52)	1.66 (0.28–6.91)
4–9	13/10.8	1.21 (0.64–2.06)	1.23 (0.62–2.22)	3/1.1	2.64 (0.55–7.73)	1.58 (0.29–5.75)	2/1.5	1.32 (0.16–4.76)	1.82 (0.19–9.11)
⩾10	9/5.0	1.80 (0.82–3.42)	1.83 (0.81–3.66)	2/0.5	3.71 (0.45–13.41)	2.22 (0.24–9.80)	0/0.7	0.00 (0.00–5.25)	0.98 (0.00–12.01)
*P for linear trend*^d^			*0.52*			*0.22*			*0.50*

CI=confidence interval; SIR=standardised incidence ratio.

a Numbers of observed (O) and expected (E) incident cancers estimated assuming the cohort experienced the same calendar year and age-specific incidence rates as the England and Wales female general population.

b Relative risk (95% confidence interval) as estimated by the SIR ratio.

c Treatments are not mutually exclusive.

d Estimated only among those women who were prescribed the treatment.
